# Recent advances in carbocupration of α-heterosubstituted alkynes

**DOI:** 10.3762/bjoc.6.77

**Published:** 2010-07-15

**Authors:** Ahmad Basheer, Ilan Marek

**Affiliations:** 1 The Mallat Family Laboratory of Organic Chemistry, Schulich Faculty of Chemistry and the Lise Meitner-Minerva Center for Computational Quantum Chemistry, Technion-Israel Institute of Technology, Haifa 32000, Israel

**Keywords:** alkynes, carbocupration, enamides, regioselectivity, stereoselectivity, syn-addition, vinylcopper, ynamides, ynol ether

## Abstract

Carbocupration of α-heterosubstituted alkynes leads to the formation of stereodefined functionalized vinyl copper species as single isomer. Recent advances in the field show that a simple pre-association of the organometallic derivative with an additional polar functional group in the vicinity of the reaction center may completely change the stereochemical outcome of the reaction. Representative examples are given in this mini-review.

## Review

**Scheme 1:**
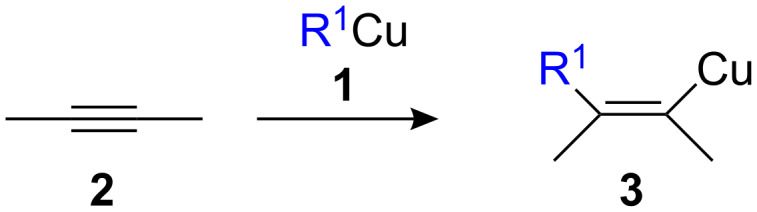
General scheme for the carbocupration reaction.

The addition of a carbon-metal bond of an organometallic species to an alkyne (carbometalation reaction) is an extremely useful reaction for the preparation of polysubstituted stereodefined alkenyl metal derivatives. When the organometallic species involved is an organocopper reagent, the reaction is termed carbocupration. To be synthetically useful, the new organocopper **3** must have a reactivity different from that of **1** in order to avoid oligomerization of the carbometalated product **3** (Scheme 1) [1–3].

**Scheme 2:**
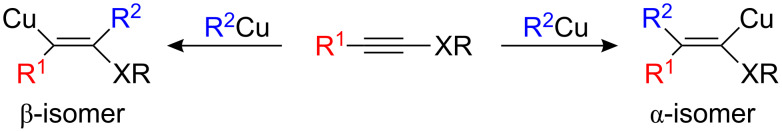
Regioselectivity in the carbocupration reaction.

For most carbocupration reactions, a strict *syn*-addition prevails but the regioselectivity is usually dependent of the substitution pattern of the alkyne [4]. Indeed, the presence of a donor or acceptor groups in close proximity to the acetylenic moiety can influence the regioselectivity of the addition and different isomers could be formed [5–7]. The most pronounced effect concerns the intermolecular addition of an organocopper to α-heterosubstituted alkynes such as *O*-, *N*-, *P*-, *S*-, *Sn*-, *Si*-substituted alkynes. In this review the earlier results of carbocupration reactions will be first briefly summarized to underscore the issue of regioselectivity, and then the most recent examples will be discussed in detail (Scheme 2). Two isomers could be obtained according to the nature of the heteroatom XR; the α-isomer (or *linear* product after hydrolysis when R^1^ = H) in which the copper atom adds to the carbon bearing the heteroatom, or the β-isomer (or branched adduct after hydrolysis when R^1^ = H) when the copper atom is in a β-position with respect to the heteroatom.

### Alkoxy-substituted alkynes

**Scheme 3:**
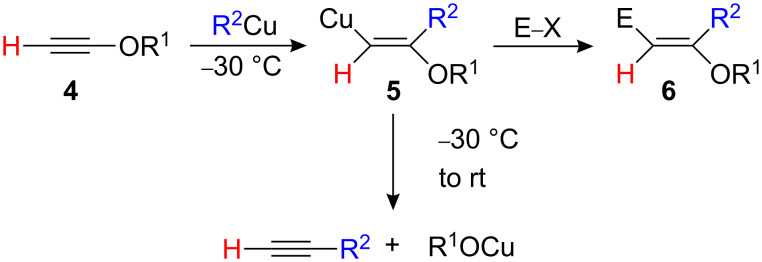
Carbocupration of α-alkoxyalkynes.

For alkoxy-substituted acetylene **4a**, R^2^Cu•MgBr_2_, R^2^Cu•LiI or R^2^_2_CuLi react equally well to give the branched products **6** in good to excellent yields [8–10]. The selectivity can be explained by the electrostatic charge distribution in the alkynes [11–13]. Particularly interesting is the addition of trimethylsilylmethyl copper that leads to the preparation of functionalized enol ethers possessing an allylsilane moiety [14–16]. However, the carbometalated product **5** must be kept at low temperature to avoid any elimination reaction (Scheme 3).

**Scheme 4:**
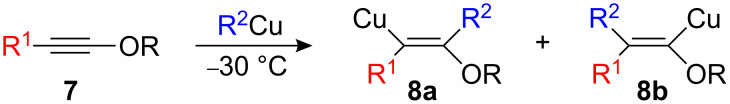
Carbocupration of substituted α-alkoxyalkynes.

Such a strategy was employed in the two-directional synthesis of the F-J fragment of Gambieric Acid [17]. On the other hand, when substituted ynol ether **7** (R and R^1^ = alkyl) was treated with the same organocopper reagents under the same conditions, the two substituents flanking the triple bond have opposite effects and two isomers **8a** and **8b** were formed in an equimolar ratio (Scheme 4) [8–9].

**Scheme 5:**
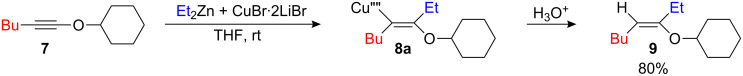
Formation of the branched isomer.

Interestingly, when Et_2_Zn is treated with CuI in a 1:1 ratio, the carbocupration reaction of substituted alkynyl ether **7** proceeds at room temperature to give mainly the β-isomer **8a** (**8a**/**8b** = 97/3) in 67% yield [18]. When CuBr was used instead of CuI, the yield was better although the regioselectivity of the reaction was slightly decreased (**8a**/**8b** = 93/7 in 82% yield). The best compromise was to use Et_2_Zn with a soluble copper salts such as CuBr•2 LiBr or CuCN•2LiBr (or LiCl). In both cases, the β-isomer **8a** was obtained in quantitative yields with an excellent regioisomeric ratio (**8a**/**8b** = 98/2, Scheme 5).

The nature of the alkyl group of the dialkylzinc species can be broadened since it could also be prepared in-situ by a transmetalation reaction of RMgX with half equivalent of a zinc salt. The scope of the reaction was extended to include various dialkylzinc species containing not only the “challenging” methyl group but also various primary and even some secondary aliphatic groups. In all cases, excellent ratios for the formation of the β-isomer **8a** were obtained.

**Scheme 6:**
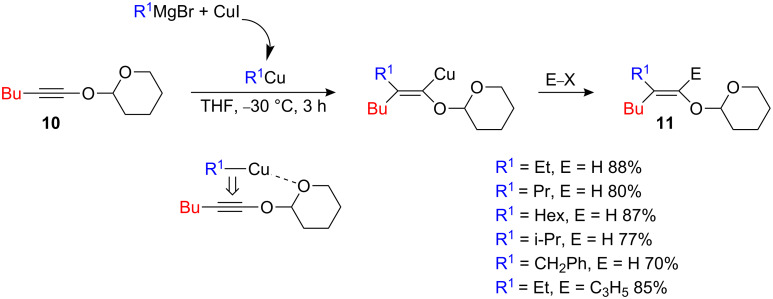
Formation of the linear isomer.

To extend the power of carbocupration reaction for useful synthetic transformations, it would be important to prepare selectively the α-isomer **8b** as well (Scheme 4) [18]. As carbocupration of **7** leads to either variable amounts of the α- and β-isomers (Scheme 4) or mainly to the β-isomer (Scheme 5), the exclusive formation of the α-isomer could only be achieved via pre-association of the organometallic derivative with an additional polar functional group in the vicinity of the reaction center. Such interactions between substrate and reagent, which are attractive rather than repulsive in nature, frequently provide a stereochemical outcome that is opposite to that predicted on the basis of electronic or steric effects alone [6–7]. Therefore acetylenic ether **10** possessing an additional chelating group was prepared and the regiochemistry of the carbometalation investigated as shown in Scheme 6 [8].

The addition of various organocopper species resulting from the addition of 1 equivalent of alkylmagnesium bromide [R^1^ = primary, secondary and benzyl groups] to 1 equivalent of copper salt leads, after reaction with electrophiles, to the unique formation of the linear adducts **11** in excellent isolated yields. The intramolecular chelation of the organocopper by the oxygen of the THP completely reversed the regiochemistry of the carbocupration reaction [8].

**Scheme 7:**
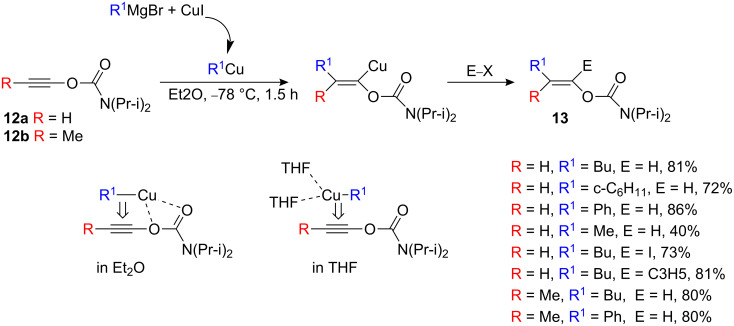
Carbocupration of O-alkynyl carbamates.

Ethynyl carbamate is also an oxy-substituted acetylene (and therefore should lead to the branched product). However, the electron-withdrawing group properties of the carbamoyl group combined with its strong ability to coordinate organometallic derivatives have a major directing effect and favor formation of the α-isomer (Scheme 7) [19].

Thus, when alkynyl carbamates **12a,b** are added to organocopper derivatives RCu•MgBr_2_ in Et_2_O at −78 °C for only 90 min, the expected linear products **13** are obtained after hydrolysis exclusively as (*E*)-isomers. The reaction is extremely rapid (90 min at −78 °C) and primary as well as secondary alkylcopper reagents lead, after hydrolysis, smoothly to pure (*E*)-isomers. It is interesting to note that whilst phenyl and methyl copper species are known to be very sluggish in carbocupration reactions [1], the carbamate moiety allows reactions with these groups to proceed in good yields. Although much more difficult to prepare, substituted alkynyl derivatives such as propynyl carbamate **12b** also reacts easily with organocopper species to afford stereospecifically the trisubstituted enol carbamates as single isomers. It is important to note that the nature of solvent plays a critical role in the regioselectivity of this reaction. Diethyl ether leads to the linear product, whereas THF gives the branched product as the major reaction product (branched/linear 80/20, Scheme 7).

Copper-catalyzed carbomagnesiation could even further increase the efficiency of these reactions. Thus, when ethynyl carbamate **12a** was added to a stoichiometric amount of alkylmagnesium halide in Et_2_O in the presence of 10 mol % CuI, the corresponding (*E*)-substituted alkene was obtained in 70% isolated yield after hydrolysis [9]. The addition of a typical electrophile to vinylmagnesium halide species such as aldehydes, is therefore now possible. Interestingly, this copper-catalyzed carbomagnesiation reaction proceeds at slightly higher temperatures than the stoichiometric process (−40 instead of −78 °C), which can be attributed to a slow copper to magnesium contrathermodynamic transmetalation reaction, probably due to a strong intramolecular chelation of the sp^2^ organometallic derivative by the carbamate moiety.

### N-substituted alkynes

**Scheme 8:**
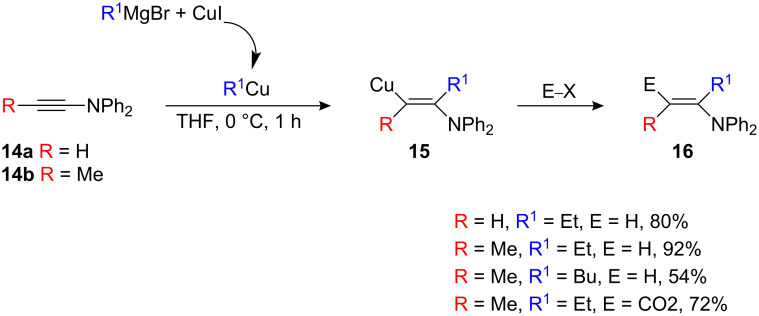
Carbocupration of ynamines.

Nitrogen-substituted alkynes (ynamine) undergo carbocupration to give the branched product **16** in good yields and as single isomers irrespective of whatever substitutents are present on the starting alkyne **14 a,b** (R = H, Me, Scheme 8) [8–9].

**Scheme 9:**
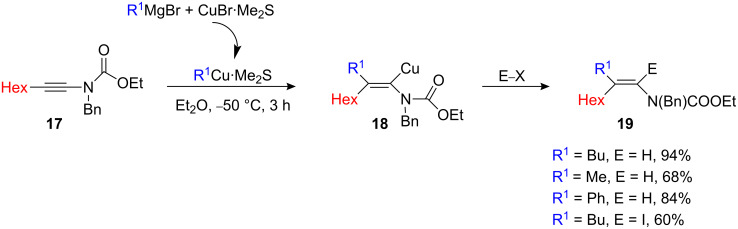
Carbocupration of ynamide.

The vinylic organocopper **15** is more stable than the corresponding β-metalated enol ether **5a**: **5a** undergoes β-elimination at −20 °C (Scheme 3) whilst β-elimination of **15** proceeds only at +20 °C. However, the resulting enamine **16** was found to be unstable and easily underwent isomerization. The formation of a single branched regioisomer **15** in the carbometalation reaction of **14** can be rationalized through the charge distribution in the alkyne (electron donating nitrogen atom induces polarization) [11–13]. To obtain the opposite regioisomer for nitrogen-substituted alkynes (linear isomer), it is essential to overcome the effect of the electron-donating heteroatom. Therefore, ynamides **17** (considerably more robust than classical ynamines) [20–21] was found to be the best candidates since they combine an electron withdrawing substituent with a chelating moiety (Scheme 9) that could possibly reverse the regioselectivity of the carbometalation. Indeed, the carbocupration reaction of **17** with an organocopper reagent gave the corresponding vinylic organocopper intermediate **18** at low temperature, which can be trapped with electrophiles. Only the linear isomer was formed in the process [22].

**Scheme 10:**
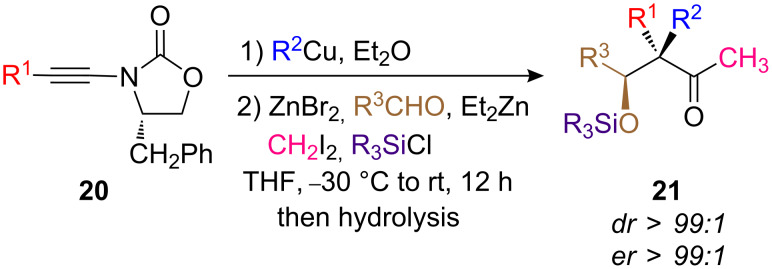
Formation of aldol products possessing stereogenic quaternary carbon centers.

Organocopper generated from CuBr•Me_2_S has a beneficial effect in the conversion since a better yield was obtained (CuI leads to roughly 20% less). Copper-catalyzed carbomagnesiation leads to the same stereochemistry although this transformation requires a slightly higher temperature (−30 °C to rt). Et_2_O is the solvent of choice for this transformation since THF leads to the branched isomer as the major product (branched/linear: 82/18), again showing that the chelation effect plays a major role in the regiochemistry of the carbometalation. The carbometalation of enantiomerically enriched cyclic ynamide **20** was recently used in a single-pot operation as a new entry to aldol products **21** possessing quaternary stereocenters (Scheme 10) [23].

**Scheme 11:**
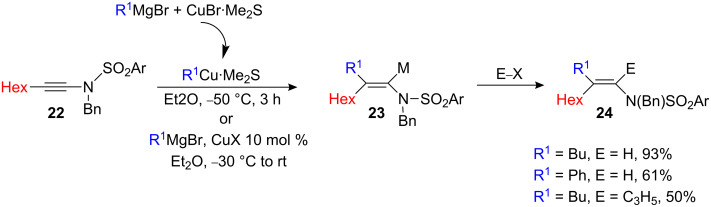
Carbocupration of alkynyl sulfonamide.

Sulfonyl-substituted ynamide **22** has also been investigated in carbocupration and copper-catalyzed carbomagnesiation reactions (Scheme 11) [22]. Irrespective of the conditions, the carbometalation reaction on sulfonyl-substituted ynamide **22** is slower than that with ynamide **17**. In the stoichiometric version, the reaction proceeds smoothly for the addition of alkyl and phenyl substituents but the yield is much lower for the addition of methyl (30% yield, not indicated in Scheme 11).

**Scheme 12:**
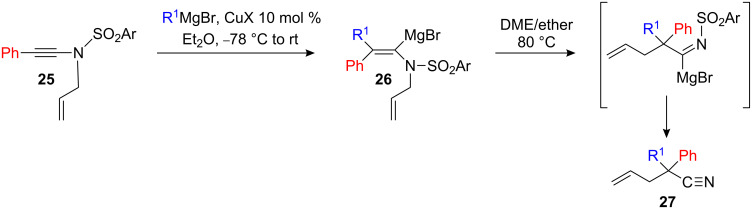
Tandem carbocupration-sigmatropic rearrangement.

Even when phenylethynylarene sulfonamide **25** is used as starting material, a single regioisomer **26** was obtained in the copper-catalyzed carbomagnesiation reaction despite the possible formation of a more stable benzylic organometallic that could have changed the regioselectivity of the carbometalation [24]. Interestingly, when an allyl group is on the nitrogen atom, the carbometalated product **26** undergoes a subsequent thermal [3,3]-sigmatropic rearrangement to give the corresponding nitrile **27**. The presence of the organomagnesium group on **26** is essential for the rearrangement to proceed in good yield (Scheme 12).

**Scheme 13:**
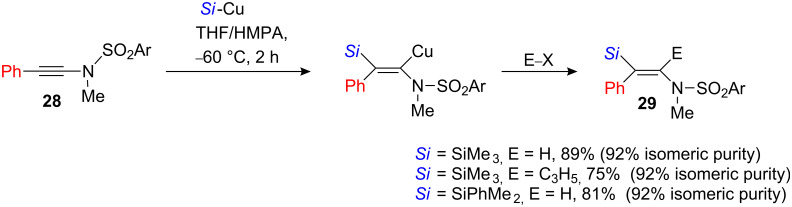
Silylcupration of alkynyl sulfonamides.

The silylcupration of *N*-1-alkynylsulfonylamides **28** led to the desired vinylsilane adduct **29**. However, the reaction was not completely stereoselective (Scheme 13) [25].

Although not a copper-mediated transformation, the rhodium-catalyzed carbozincation of ynamides should be noted since the regioselectivities were, in general, high (>19:1), though in some cases somewhat diminished with Me_2_Zn (5:1) [26–27]. The rhodium-catalyzed annulation of ynamides with arylboron compounds containing an aldehyde or a ketone moiety have also been developed and lead to functionalized 2-amidoindenes with good levels of regioselectivity [28].

### P-substituted alkynes

**Scheme 14:**
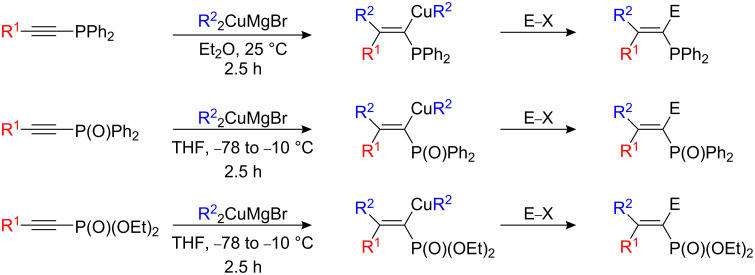
Carbocupration of P-substituted alkynes.

Stereo- and regioselective carbometalation of 1-alkynylphosphines with magnesium dibutylcuprate (Bu_2_CuMgBr) leads to diphenyl(butylethynyl)phosphine in good isolated yields [29–30]. The addition of various electrophiles (with the exception of aldehydes and methyl iodide) such as allylbromide, benzoyl chloride) was successful (Scheme 14).

**Scheme 15:**
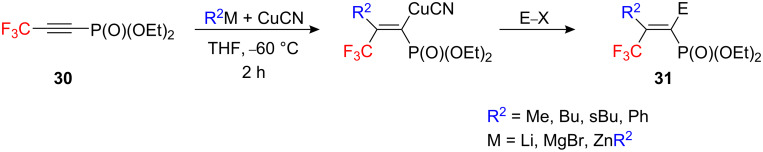
Carbocupration of alkynylphosphonates.

The two alkyl groups on the cuprate reagent could be eventually transferred when the reaction was performed in THF instead of Et_2_O. The same trend was found for the carbocupration of alkynyl phosphine oxide [31] and alkynyl phosphonates [32]. In the latter case, when R^2^ = Ph, only one isomer was obtained at −10 °C but a mixture was formed with the anti adduct as the major product at room temperature. When R^2^ = *t*-Bu, anti addition was observed exclusively. Fluorinated alkynylphosphonates **30** can also be carbometalated with various organometallic reagents as summarized in Scheme 15 [33]. When RCuCNLi (R = Me, Bu, sBu, Ph) was added to **30**, a single isomer of the alkenylphosphonate **31** was obtained. Similarly, alkyl and aryl Grignard reagents as well as dialkylzinc compounds are transmetalated to their copper species and react regio- and stereoselectively with **30** (Scheme 15) [33].

### S-substituted alkynes

**Scheme 16:**
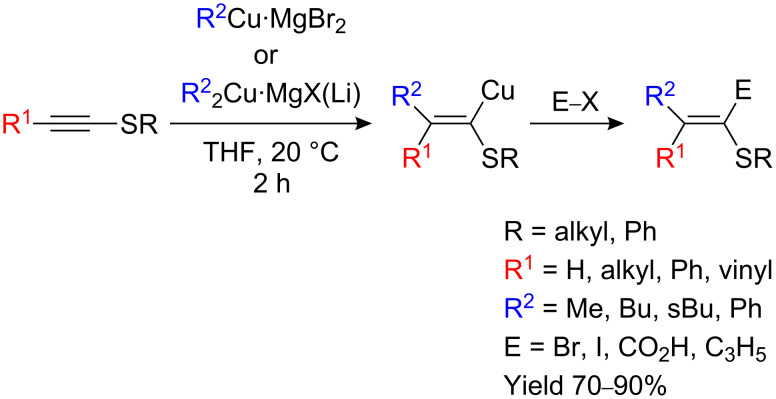
Carbocupration of thioalkynes.

When alkylthioacetylenes are treated with an organocopper reagent such as RCu•MgX_2_ or R_2_Cu•MgX(Li) in THF, the linear product is formed exclusively and is remarkably stable even at +60 °C (Scheme 16) [8–9,34].

**Scheme 17:**
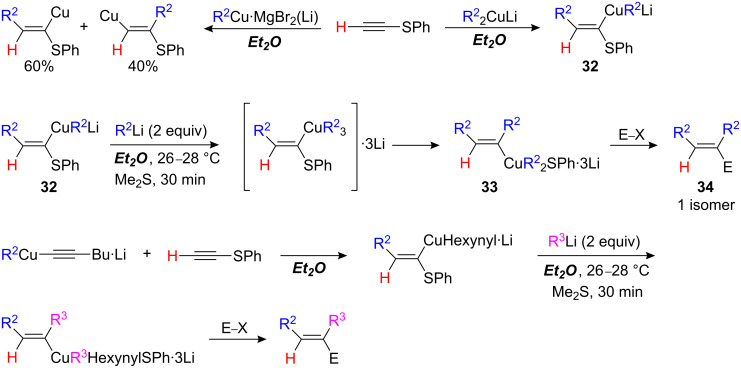
Tandem carbocupration-1,2-metalate rearrangement.

Surprisingly, in less polar solvents such as Et_2_O, two isomers are obtained when an organocopper RCu is added to phenylthioethyne. Only cuprate R_2_CuLi leads to the linear isomers in good yield (Scheme 17) [35]. Addition of a cuprate to 1-substituted alkynylsulfides does not lead to addition but to substitution at sulfur and the formation of a metalated alkyne.

When **32** is treated with an excess of R^2^Li, a 1,2-metalate rearrangement occurs to give a new organocuprate reagent **33** with a copper now *trans* to the initial alkyl group [35]. After re-cooling the reaction mixture to −40 °C, the addition of electrophiles affords functionalized trisubstituted alkenes **34** as single isomers. In every case examined, the stereochemistry of the formed polysubstituted alkenes corresponds to >99% stereoselectivity. The kinetics of the intramolecular substitution is dependent on the reaction temperature. If the temperature is too low (i.e. −10 °C) no rearrangement takes place; at 0 °C, rearrangement occurs but a 60–70% yield is obtained only after a considerable period of time (48 h), whilst at 26 °C to 28 °C, the *cis* vinyl alkyl cuprate is transformed into the *trans* vinyl alkyl cuprate in 30 min. To increase the stability of the vinyl organometallic towards the effect of temperature, Me_2_S was introduced before the rearrangement step. The use of an acetylenic group as a non-transferable or dummy ligand in the initial organocopper reagent, allows the selective transfer of two different alkyl groups according to Scheme 17.

**Scheme 18:**
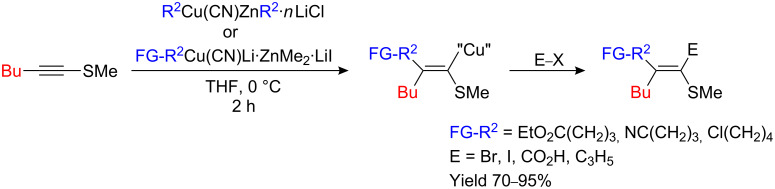
Carbocupration with functionalized organocopper species.

Dialkylzinc or polyfunctionalized alkylzinc halides (FG-RZnI) are transmetalated with CuCN•*n*LiCl (*n* = 1, 2) or with Me_2_Cu(CN)Li_2_ in THF, respectively and lead to copper reagents tentatively formulated as RCu(CN)ZnR•*n*LiCl and FG-RCu(CN)Li•ZnMe_2_•LiI. These organocopper reagents react smoothly with 1-(methylthio)-1-hexyne to yield stereochemically pure linear alkenylcopper species that can react with a large variety of electrophiles (Scheme 18) [36].

**Scheme 19:**
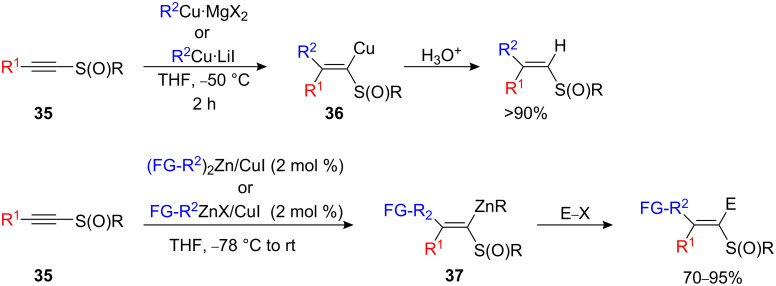
Carbocupration of alkynyl sulfoxides.

The regio- and stereospecific carbocupration of higher oxidation states of sulfur (i.e. sulfoxides **35**) with organocopper derivatives (R^2^Cu•MgX_2_ or R^2^Cu•LiX) rapidly provides the corresponding metalated β,β-dialkylated ethylenic sulfoxides **36** in quantitative yields (Scheme 19) [37–40] that can be further used in organic synthesis [41–43]. However, to obtain functionalized dialkylated ethylenic sulfoxide species, the addition of functionalized organozinc species is required. Upon treatment with (FG-R^2^)_2_Zn (2 equiv) or FG-R^2^ZnX in the presence of a catalytic amount of CuI (2 mol %), 1-alkynyl sulfoxide **35** undergoes carbozincation to give the functionalized vinylic sulfoxide (*Z*)-**37** in excellent yield. The reaction proceeds in a *syn*-selective fashion to give only the linear isomer (Scheme 19) [44–45].

The fact that α-sulfinyl vinylcopper and zinc intermediates, **36** and **37** respectively, retain their geometry at room temperature is in sharp contrast to α-sulfinyl vinyllithium- or magnesium species that isomerize to the thermodynamically more stable geometric isomer at low temperature. When an enantiomerically pure sulfoxide was used as starting material, no racemization was detected [40].

**Scheme 20:**
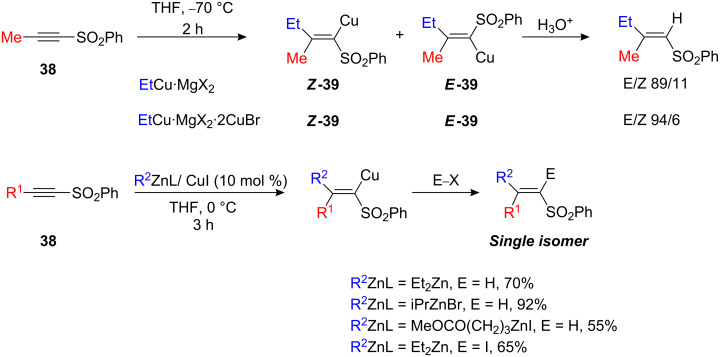
Carbocupration of alkynyl sulfones.

1-Alkynyl sulfones **38** are prone to addition of organocopper reagents. However, the stereochemistry of the resulting vinyl sulfones **39** appear to be dependent on the relative amounts of copper(I) bromide as well as the Grignard from which the organocopper compound was prepared (Scheme 20) [46–50]. Excess of copper salt provides a better stereoisomeric ratio (>90%) [46]. Only the addition of *t*-BuCu gave a single stereoisomer [51]. In contrast, the copper-catalyzed carbozincation reaction led to a single stereoisomer via *syn* addition under mild conditions in either THF or Et_2_O (Scheme 20) and the reaction could be expanded to incorporate a large variety of functionalized alkylzinc bromide and dialkylzinc species [52].

**Scheme 21:**
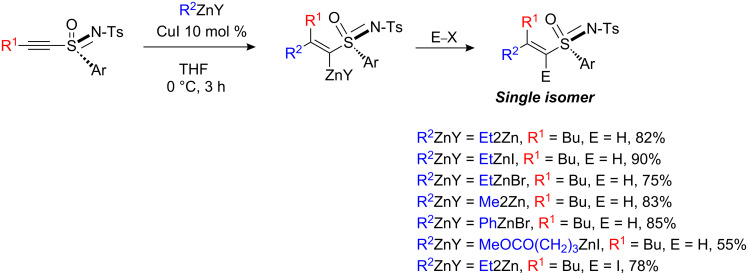
Carbocupration of alkynyl sulfoximines.

Copper-catalyzed carbozincation could also be performed under very different conditions (toluene, reflux) and a single isomer was still obtained [53]. 1-Alkynyl sulfoximines behave similarly to alkynyl sulfones and the addition of an organocopper reagent usually leads to two isomers in variable amounts. Copper-catalyzed carbozincation to give a single regio- and stereoisomer in excellent yield is possible only for few representative examples (Scheme 21) [52].

### Si-substituted alkynes

**Scheme 22:**
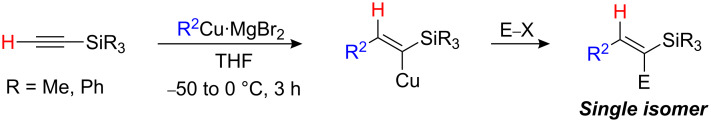
Carbocupration of alkynylsilanes.

Alkylcopper reagents, prepared from RMgBr and CuBr react with trimethylsilylacetylene [54] and triarylsilylacetylene [55] to give regio- and stereoselectively 1-trimethyl(aryl)silyl-1-(*Z*)-alkenyl copper adducts (Scheme 22). Hydrolysis, alkylation, acylation, halogenation and stannylation proceed stereoselectively to afford synthetically useful intermediates [56–57]. Under similar conditions, 1-substituted alkynylsilanes do not undergo the carbometalation reaction.

**Scheme 23:**
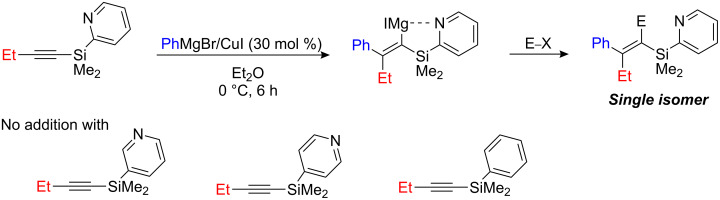
Carbocupration of functionalized alkynylsilanes.

Only when 1-substituted-2-pyridylsilylalkyne is used, does the copper-catalyzed carbomagnesiation reaction proceed with the formation of the corresponding Grignard reagent. To prove the involvement of the pyridyl coordination, the 3- and 4-pyridylsilyl- and phenylsilyl substituted substrates were prepared and treated under similar reaction conditions. In all cases, no carbomagnesiation occurred, which proves that the directing group assistance is essential for both reactivity and regioselectivity (Scheme 23).

During these investigations, it was found that the amount of CuI employed has a significant influence on the efficiency of carbomagnesiation. The reaction with PhMgI (1.0 equiv) using various amounts of CuI (0 to 100 mol %) was studied. No addition occurred in the absence of CuI and the yield was found to increase with increasing amounts of CuI and reached a maximum (90%) at 30 mol % based on alkynylsilane and PhMgI [58]. However, further increases in the amount of CuI added resulted in lower yields. In particular, when an equimolar amount of CuI was employed, there was no addition, which suggests that the carbometalation does not proceed through an organocopper species but rather via an organocuprate species. In line with this assumption, the carbometalated adduct was obtained in 83% yield even when using an equimolar amount of CuI with 2.0 equiv of PhMgI. Moreover, yields are drastically affected by the nature of the aryl Grignard reagent used (PhMgI, 74%; PhMgBr, 27%; PhMgCl, 0%; Ph_2_Mg, 0%): other Grignard derivatives were not investigated in this reaction [59].

**Scheme 24:**
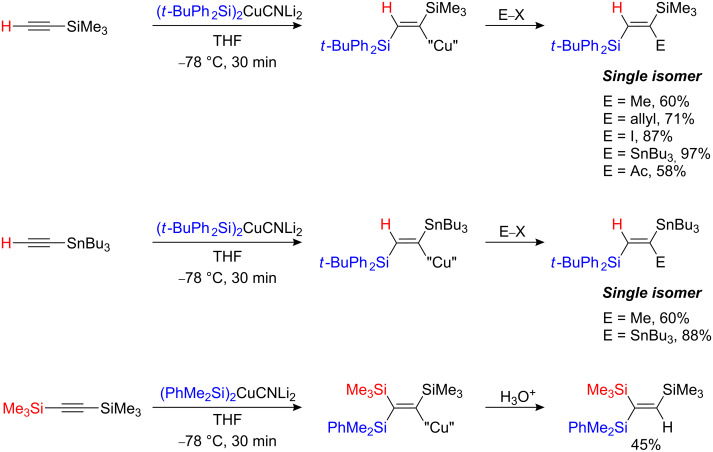
Silyl- and stannyl cupration of silyl- and stannylalkynes.

Although not strictly related to the field of carbocupration reaction, the silylcupration of ethynylsilane and tributylstannylacetylene lead to the formation of 1,2-bis(silylated) and stannylated vinyl copper species, respectively. These organometallic species react with a wide range of electrophiles to afford regio- and stereodefined *vic*- and *gem*-silyl (and tin) trisubstituted alkenes (Scheme 24). Even the deactivated bis-trimethylsilylacetylene reacted the dimethylphenylsilylcuprate reagent in presence of TMEDA to afford the trisilylalkene in moderate yield [60].

## Conclusion

In conclusion, the intermolecular carbocupration of α-heterosubstituted alkynes can now be easily manipulated to prepare either linear or branched isomers as single regioisomers. A stereodirecting effect through intramolecular chelation plays a fundamental role in carbocupration reactions. *O*- and *N*-substituted alkynes give the β-isomer due to the mesomeric effect of the heteroatom, except when an extra chelating moiety is present such as in alkynyl-O-carbamates, THPoxy-alkynes, and ynamides. In these cases, the opposite α-isomers are formed. *P*-, *S*- and *Si*-substituted alkynes all give the α-isomers. This effect can even be reinforced when an additional chelating unit is present (pyridylSi, phosphonates, sulfoxides, sulfones, sulfoximines). The resulting vinyl copper species can react with a large variety of electrophiles leading to functionalized adducts in a straightforward manner that can be further modified [61–72]. No doubt more subtle variations will continue to appear to enrich the wonderful chemistry of organocopper chemistry.
